# The impact of COVID-19 on an inpatient mother and baby unit: a service evaluation

**DOI:** 10.1192/bjo.2021.833

**Published:** 2021-06-18

**Authors:** Joanna Cranshaw, Gertrude Seneviratne, Ranga Rao, Julia Ogunmuyiwa, Rebecca McMillin, Chukwuma Ntephe, Victoria Dain

**Affiliations:** South London and Maudsley NHS Foundation Trust

## Abstract

**Aims:**

Unique challenges have been faced by women in the perinatal period during the COVID-19 pandemic and the impact of this is compounded for women suffering from mental illness. This service evaluation looked at different aspects of the treatment pathway on a specialist inpatient psychiatric Mother and Baby Unit during the pandemic to identify what changes occurred.

**Method:**

Data were collected for all admissions to the unit between January 2019 and October 2020, with the beginning of the pandemic being defined as on or after 1st March 2020. Information was collected retrospectively from electronic clinical notes on ethnicity, length of stay, diagnosis, mental health act use and restrictive practice, medication, psychology, occupational therapy and social services involvement.

**Result:**

There were 114 admissions to the MBU during the study period. 4 were parenting assessments rather than acute psychiatric admissions and were excluded from the analysis, giving a sample of 110 women. 58% (62/110) were classed as “pre-pandemic” and 43.6% (48/110) were “during pandemic”. 95.45% (105/110) of women were postpartum 4.55% (5/110) were pregnant. Mean length of stay was shorter during the pandemic at 44 days, compared to 61 pre-pandemic. There was greater use of the mental health act during the pandemic: only 43.75% of patients were informal throughout admission, compared to 70.97% pre-pandemic. Mean duration of detention was shorter at 25 days (32 pre-pandemic). Psychotic illness made up a greater proportion of diagnoses during the pandemic: 56% (27/48) compared to 44% (27/62) pre-pandemic. The next most common diagnostic group was mood and anxiety disorders, which made up 29% (14/48) of diagnoses during the pandemic, but 43% (27/62) pre-pandemic. Outcomes as measured using the Health of the Nation Outcome Scale showed a mean improvement between admission and discharge of 6.65, compared to 5.15 pre-pandemic. HONOS scores were higher on admission during the pandemic (12.83, vs 10.88), suggesting a higher level of acuity.

**Conclusion:**

During the COVID-19 pandemic on this Mother and Baby Unit, length of stay was shorter, a greater proportion of patients were detained under the mental health act (although length of detention was shorter) and psychotic illness was more prevalent. This study demonstrates that there were differences in this perinatal inpatient population during the pandemic and this may be a reflection on the wider impact of COVID-19 on perinatal mental health.

